# Editorial: Innate immune pathways as targets for developing therapeutic intervention against human cancers

**DOI:** 10.3389/fimmu.2025.1595279

**Published:** 2025-05-09

**Authors:** Fabio Grizzi, Miguel Martin-Perez, Geeta Rai, Abdullah Farhan ul Haque Saeed, Ayush Raman, Devivasha Bordoloi

**Affiliations:** ^1^ Department of Immunology and Inflammation, IRCCS Humanitas Research Hospital, Milan, Italy; ^2^ Department of Biomedical Sciences, Humanitas University, Milan, Italy; ^3^ Department of Cell Biology, Physiology and Immunology, University of Barcelona, Barcelona, Spain; ^4^ Department of Molecular & Human Genetics, Institute of Science, Banaras Hindu University, Varanasi, India; ^5^ Department of Internal Medicine, University of Michigan, Ann Arbor, MI, United States; ^6^ National Cancer Institute, NIH, Bethesda, MD, United States; ^7^ Department of Biological Sciences, Indian Institute of Science Education and Research (IISER) Bhopal, Bhopal, India

**Keywords:** innate immunity, innate immune pathway, cancer, therapy, drugs

The innate immune system serves as the body’s initial barrier of defense against invading pathogens, ensuring a rapid response to infections (1). Unlike the adaptive immune system, which requires time to develop a targeted response, the innate immune system provides a broad yet immediate defense against pathogens (2, 3). It detects pathogens using specialized receptors, processes the information through signaling pathways, and then triggers a targeted response, including the activation of the inflammatory response. Inflammation occurs when innate immune cells recognize infection or tissue damage (4).

**Figure 1:**
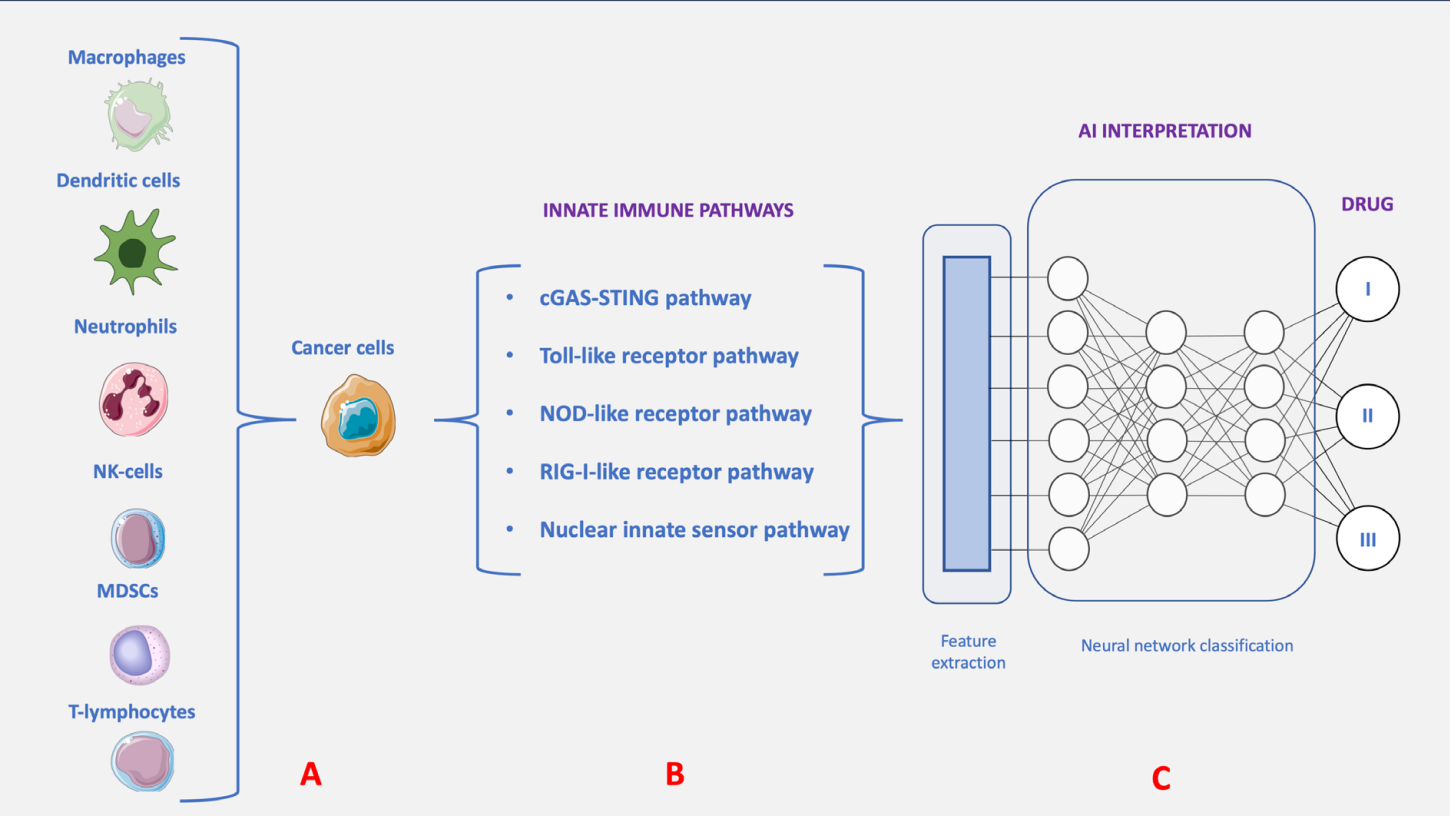
The tumor immune microenvironment (TME) includes macrophages, dendritic cells, T lymphocytes, neutrophils, myeloid-derived suppressor cells, and natural killer cells, forming a network with both pro- and anti-tumor effects. The TME influences immune cell differentiation and polarization, often promoting a pro-tumor state. Cell-to-cell communication between the immune system and tumors shapes tumor development, progression, and treatment response **(A)**. Stressors like drugs, immune cytotoxicity, and hypoxia cause tumor cell leakage or death, releasing DAMPs into the TME, detected by PRRs to activate innate immune pathways **(B)**. Antigen-presenting cells amplify DAMP production by engulfing tumor cells. Analyzing innate immune pathways is vital for clinical cancer treatments, requiring an interdisciplinary approach and leveraging AI to enhance predictive modeling, improving drug efficacy and accelerating discovery **(C)**.

The “innate immune pathway” is attracting growing interest in cancer treatment because of its broad expression across different cell types, such as immune, tumor, and stromal cells (5). The innate immune pathway network varies across different cell types, being controlled by cell-specific regulatory mechanisms that result in diverse functional responses to identical stimuli (**Figure 1**). This modulation determines whether immune responses support or inhibit tumor growth. However, disturbances in intracellular signaling within immune cells, along with adaptive changes in tumor cells in the microenvironment, frequently compromise innate immune pathways, hindering their proper function. Understanding and strategically modulating these pathways in the Tumor microenvironment (TME) is essential for leveraging them in cancer therapy.

The “danger theory” explains why strong immune responses arise despite the absence of microbial components (6). In response to trauma, ischemia, and cellular damage or death, molecules that typically serve non-immunological functions within cells are released, secreted, or exposed on the cell surface, triggering an immune response independent of infection. These molecules, later identified as damage-associated molecular patterns (DAMPs), are crucial in triggering innate immune responses and promoting the production of pro-inflammatory cytokines and interferons (IFNs). Molecules such as DAMPs, which stimulate innate immune signaling, are abundant in the TME, reinforcing the therapeutic potential of targeting these pathways (7). DAMPs play a crucial role in activating innate immune mechanisms in cancer. Among these mechanisms, aberrant DNA recognition via the cGAS - STING pathway is particularly significant for detecting transformed cells, both under normal conditions and after cancer treatment (8). Cyclic GMP-AMP (cGAMP) synthase (cGAS) functions as a cytosolic DNA sensor that activates the stimulator of interferon genes (STING) protein, initiating a defensive immunological activation against DNA-based pathogens and strengthening anti-cancer immune activity (9, 10). The toll-like receptors (TLRs) family are expressed across various immune cell types and recognize diverse pathogen-associated molecular patterns (PAMPs) and DAMPs, including DNA, RNA, and lipopolysaccharides (LPS). Tumor cells also express multiple TLRs, and their activation can autonomously trigger cell death through different pathways (11). Activation of TLRs can reverse the immunosuppressive effects of tumor-associated cells by modulating metabolism, making them promising targets for cancer immunotherapy. The NOD-like receptor (NLR) pathway consists of cytosolic sensors called Nucleotide Oligomerization Domain (NOD)-like receptors, which are crucial for detecting infections and controlling autoinflammatory responses. The RIG-I-like receptor (RLR) pathway comprises cytoplasmic sensors that recognize viral RNA, including Retinoic Acid-Inducible Gene 1 (RIG-I), Melanoma Differentiation-Associated Factor 5 (MDA5), and Laboratory of Genetics and Physiology 2 (LGP2) (12). RLR activation primarily engages the NF-κB pathway and promotes apoptosis. These receptors also influence tumorigenesis, with studies showing that reduced RIG-I expression facilitates the development of hepatocellular carcinoma (HCC) (13). Recently, several nuclear molecules have been identified as “innate sensors” that activate the immune pathway. These include Z-DNA binding protein 1 (ZBP1), Scaffold-attachment-factor A (SAFA), and Heterogeneous Nuclear Ribonucleoprotein A2B1 (hnRNPA2B1). Other key molecules include Interferon gamma-inducible protein 16 (IFI16) and Non-POU domain-containing octamer-binding protein (NONO). Although these targeted drugs have demonstrated significant efficacy in preclinical trials, their success in clinical settings has been only marginal. Identifying the factors behind the inconsistent therapeutic effectiveness of these targeted drugs is critical and requires further investigation (14).

This Research Topic explores recent advancements in innate immune signaling pathways that are related to both the protection and pathogenesis of human cancers. Technological advancements, new methodologies, and the development of novel knowledge and fundamental insights, alongside the exploration of new targets and therapeutics, are expected to further strengthen ongoing research in this area. The Research Topic presents a comprehensive selection of articles that address the immunobiological relevance of innate immune pathways in the pathogenesis of various human cancer subtypes and the host immune response to cancer. It also highlights the therapeutic potential of innate signaling-directed chemo- and immunotherapeutic interventions in human cancers.

Xue et al. investigate the complex interplay of cGAS-STING signaling in chronic hepatitis, alcoholic liver disease (ALD), metabolic dysfunction-associated steatotic liver disease (MASLD), and HCC, discussing its potential as a therapeutic target. Emerging evidence indicates that cGAS-STING signaling is crucial for maintaining liver homeostasis and contributes to the onset and course of various liver diseases. The authors offer a detailed analysis of the cGAS-STING pathway, with a focus on its signaling cascade and involvement in several major liver diseases.

In HCC, cGAS-STING-targeted strategies include nanomaterial-based delivery of STING agonists, combining radiofrequency ablation or radiotherapy to enhance pathway activation. Modulating cGAS-STING may also offer treatment options for chronic viral hepatitis, MASLD, and ALD by boosting antiviral defenses or reducing inflammation. This highlights the pathway’s complex role in liver diseases and the need for further research to realize its therapeutic potential.

Growing evidence highlights the cGAS-STING pathway’s key role in tumor immunity, with STING agonists enhancing immunotherapy efficacy and reducing resistance. However, this pathway can both support anti-tumor responses and promote immunosuppression. Immunosuppressive cells like M2 macrophages, myeloid-derived suppressor cells, and regulatory T cells in the TME contribute to tumor escape and limit immunotherapy success.

Zhang et al. offer an in-depth review of cGAS-STING activation, its immune functions, and its key role in immune evasion driven by the immunosuppressive TME. They also outline key immunotherapeutic approaches linked to this pathway and discuss potential enhancements to improve their effectiveness, offering important insights for future clinical use.

Recent studies highlight intra-tumoral delivery of TLR ligands as a promising way to trigger local immune responses and enhance antitumor immunity. However, their rapid spread from the TME limits efficacy and raises toxicity concerns. Kim et al. investigate intra-tumoral delivery of mRNA encoding UNE-C1, a TLR2/6 ligand recognized for its efficacy and low toxicity. Their findings demonstrate that UNE-C1 triggers immunogenic cell death through autocrine signaling, mediated by DAMP release via TLR2 activation. Sensitivity to this effect depends on TLR2 and Fas-associated death domain expression in cancer cells. UNE-C1 also activates dendritic cells via TLR2, priming CD8^+^ T cells essential for tumor regression. These findings support intra-tumoral mRNA delivery of UNE-C1 as a promising antitumor strategy.

Once seen mainly as acute inflammation mediators, neutrophils were initially overlooked in cancer. It is now clear they infiltrate the TME in large numbers as tumor-associated neutrophils (TANs), a diverse and adaptable immune subset. Rising interest in their roles has spurred research into TAN-targeted therapies, though clinical translation remains challenging. Xiao et al. review TAN-related studies published between 2000 and 2024, using data from the Web of Science Core Collection. They conduct bibliometric analysis and visualization with tools like Microsoft Excel, VOSviewer, CiteSpace, and R-bibliometrix. The analysis included 788 publications by 5,291 authors from 1,000 institutions in 58 countries/regions, published across 324 journals.

While China contributed the largest number of publications and hosted the top 10 institutions, the United States emerged as the leader in terms of high-quality publications and as a global center for collaboration. The analysis suggests that future research will likely concentrate on TAN heterogeneity, neutrophil extracellular traps, TAN interactions with other immune cells, and immunotherapy. This thorough bibliometric and visual analysis offers a detailed overview of the present state and conceptual foundation of TAN research, providing fresh insights for future investigations. Identifying distinct TAN subpopulations and precisely targeting key pro-tumor and anti-tumor groups presents significant potential for developing TAN-targeted immunotherapies.

Bacillus Calmette-Guérin (BCG) is the primary treatment for bladder cancer and is also used in melanoma immunotherapy (15). It modifies the TME to trigger a strong antitumor response, though the immune mechanisms are not fully understood.

The immune profile of B16-F10 murine melanoma cells was assessed by infecting them with BCG or stimulating them with agonists for various innate immune pathways, including TLRs, inflammasome, cGAS-STING, and type I IFN. B16-F10 cells responded only to type I IFN agonists, unlike bone marrow-derived macrophages (BMDMs), which produced high proinflammatory cytokines. Borges et al. confirm that BCG can infect B16-F10 cells, which then activate macrophages and spleen cells from mice in co-culture. They also create a subcutaneous B16-F10 melanoma model for intratumoral BCG treatment, comparing wild-type mice with various knockout models, including TLR2-/-, TLR3-/-, TLR4-/-, TLR7-/-, TLR3/7/9-/-, caspase 1-/-, caspase 11-/-, IL-1R-/-, cGAS-/-, STING-/-, IFNAR-/-, and MyD88-/-. *In vivo* findings show that MyD88 signaling is crucial for BCG immunotherapy to control melanoma in mice. BCG failed to induce cytokine production in co-culture with B16-F10, BMDMs, or spleen cells from MyD88-/- mice compared to wild-type controls. It also did not recruit inflammatory cells to the TME in MyD88-/- mice, impairing tumor control and IFN-γ production by T cells. Thus, MyD88 is pivotal for both innate and adaptive immune responses to BCG, enabling an effective antitumor response.

Glioma is a malignant tumor that affects the central nervous system (CNS) and, currently, effective treatment options remain scarce. Recent discoveries of cranial-meningeal channels and intracranial lymphatic vessels have provided new insights into the origins of neutrophils in the CNS. Neutrophils in the brain were thought to originate more from the skull and adjacent vertebral bone marrow.

It is now believed that neutrophils in the brain primarily originate from the bone marrow within the skull and adjacent vertebrae. Driven by chemokines, these cells traverse the blood-brain barrier, infiltrate the brain parenchyma, and migrate to the glioma TME, where interactions with tumor cells trigger phenotypic changes. Sun et al. provide a comprehensive review of the molecular mechanisms that govern neutrophil infiltration into the CNS from peripheral sources. Their work outlines the origin, functions, classification, and potential therapeutic targeting of neutrophils in the context of glioma. As key players in the immune system, neutrophils are gaining increasing recognition for their involvement in brain tumors. Further investigation into their role in cancer immunotherapy may open new avenues for developing more effective treatment strategies for cancer patients.

Numerous agonists targeting the innate immune system have been proposed, with several in clinical trials showing therapeutic potential. While research on the cGAS-STING pathway is still early, initial findings suggest it may offer effective and safe treatment options. Poly-ICLC, a co-agonist of TLR3, RIG-I, and MDA5, has shown clinical benefit in several trials. Everson et al. (16) report that autologous tumor lysate-pulsed dendritic cell vaccination combined with a TLR agonist was safe and enhanced systemic immunity, marked by increased interferon expression and immune cell activation. Clinical studies utilizing NLR agonists for tumor interventions, however, remain limited.

It is also evident that DAMPs are activators of the innate immune pathway, and therapies that increase DAMP production not only enhance activation but also prolong its effect. These approaches can reduce the required dosage of activators, minimizing adverse reactions. Additionally, the synergistic impact of combining innate immune agonists with immune checkpoint inhibitors (ICBs) in cancer treatment is now well understood mechanistically. The activation of intrinsic immune pathways can trigger specific pro-tumoral mechanisms, which may reduce or even counteract the effects of immune pathway agonists. Since the discovery of innate immune pathways, our understanding has advanced considerably, driving numerous preclinical and clinical cancer treatment trials. Despite progress, milestones in the clinical use of innate immune pathway agonists remain elusive. When used therapeutically, innate immune pathway agonists exhibit varying pharmacological effects on the same system, depending on factors like potency, dosing schedule, and concentration. Overall, targeting these pathways to reshape the TME and enhance tumor outcomes remains a highly compelling area of research. Innate immune pathway agonists exhibit variable effects, influenced by factors like potency, dosing interval, and concentration. Targeting these pathways to reshape the TME and improve tumor prognosis is a promising area of research.

It is now clear that further progress depends on cross-disciplinary collaboration spanning molecular biology, systems biology, immunology, and oncology. AI-powered models built from such integrated data can improve drug efficacy predictions and speed up development.
